# Reprocessed precise science orbits and gravity field recovery for the entire GOCE mission

**DOI:** 10.1007/s00190-023-01752-y

**Published:** 2023-06-30

**Authors:** Daniel Arnold, Thomas Grombein, Lucas Schreiter, Veerle Sterken, Adrian Jäggi

**Affiliations:** 1 Astronomical Institute, University of Bern, Sidlerstrasse 5, 3012 Bern, Switzerland; 2 Present Address: Geodetic Institute, Karlsruhe Institute of Technology (KIT), Karlsruhe, Germany; 3 Present Address: GFZ German Research Centre for Geosciences, Potsdam, Germany; 4 Present Address: Department of Physics, Institute for Particle Physics and Astrophysics, ETH Zürich, Zürich, Switzerland

**Keywords:** GOCE, Precise science orbits, Reprocessing, Ionosphere-induced artifacts, GPS-based gravity field models

## Abstract

ESA’s Gravity field and steady-state Ocean Circulation Explorer (GOCE) orbited the Earth between 2009 and 2013 for the determination of the static part of Earth’s gravity field. The GPS-derived precise science orbits (PSOs) were operationally generated by the Astronomical Institute of the University of Bern (AIUB). Due to a significantly improved understanding of remaining artifacts after the end of the GOCE mission (especially in the GOCE gradiometry data), ESA initiated a reprocessing of the entire GOCE Level 1b data in 2018. In this framework, AIUB was commissioned to recompute the GOCE reduced-dynamic and kinematic PSOs. In this paper, we report on the employed precise orbit determination methods, with a focus on measures undertaken to mitigate ionosphere-induced artifacts in the kinematic orbits and thereof derived gravity field models. With respect to the PSOs computed during the operational phase of GOCE, the reprocessed PSOs show in average a 8–9% better consistency with GPS data, 31% smaller 3-dimensional reduced-dynamic orbit overlaps, an 8% better 3-dimensional consistency between reduced-dynamic and kinematic orbits, and a 3–7% reduction of satellite laser ranging residuals. In the second part of the paper, we present results from GPS-based gravity field determinations that highlight the strong benefit of the GOCE reprocessed kinematic PSOs. Due to the applied data weighting strategy, a substantially improved quality of gravity field coefficients between degree 10 and 40 is achieved, corresponding to a remarkable reduction of ionosphere-induced artifacts along the geomagnetic equator. For a static gravity field solution covering the entire mission period, geoid height differences with respect to a superior inter-satellite ranging solution are markedly reduced (43% in terms of global RMS, compared to previous GOCE GPS-based gravity fields). Furthermore, we demonstrate that the reprocessed GOCE PSOs allow to recover long-wavelength time-variable gravity field signals (up to degree 10), comparable to information derived from GPS data of dedicated satellite missions. To this end, it is essential to take into account the GOCE common-mode accelerometer data in the gravity field recovery.

## Introduction

The Gravity Field and Steady-State Ocean Circulation Explorer (GOCE, Floberghagen et al. 2011), ESA’s first Earth explorer core mission, was launched on March 17, 2009, into a Low Earth Orbit (LEO). Equipped with an electric propulsion system for drag compensation to maintain the exceptionally low orbit altitude of ${224}-{254}~\hbox {km}$, a high-grade gradiometer, two geodetic-grade GPS receivers and three star trackers for the attitude determination, GOCE mapped Earth’s gravity field between 2009 and 2013 with an unprecedented accuracy and resolution. This resulted in various releases of static gravity field models computed by different approaches (Pail et al. 2011), in particular the direct approach (Bruinsma et al. 2014), the time-wise approach (Brockmann et al. 2014), and the space-wise approach (Reguzzoni and Tselfes 2009). To date, gravity field information derived from GOCE constitutes the main static contribution to global state-of-the-art Earth gravity field models, e.g., the GOCO models (Pail et al. 2010; Kvas et al. 2021).

Precise orbit determination (POD) for GOCE was enabled by tracking data of two dual-frequency 12-channel Lagrange GPS receivers (Zin et al. 2006). A seven-prism laser reflector array by the Russian Institute for Precision Instruments Engineering (IPIE, Shargorodsky 2002) allowed for the independent orbit validation by means of Satellite Laser Ranging (SLR, Combrinck 2010) data. Operational GOCE POD was performed in the frame of the GOCE High Level Processing Facility (HPF, Koop et al. 2007), which was implemented by the European GOCE Gravity Consortium (EGG-C). The latter consisted of 10 European institutions that were responsible for the processing of Level 1b to Level 2 GOCE data. The POD task was divided into two parts. The Rapid Science Orbits (RSOs) were computed at the Technical University of Delft (Visser et al. 2009, 2010) with a latency of less than 1 day and a 3-dimensional target accuracy of 50 cm. The Astronomical Institute of the University of Bern (AIUB) was responsible for the generation of the GOCE Level-2 Precise Science Orbits (PSOs, Bock et al. 2007, 2014) with a latency of one week and a required 1-dimensional accuracy of 2 cm (target 1 cm). Two types of PSOs were computed—a reduced-dynamic (Jäggi et al. 2006) and a kinematic (Švehla and Rothacher 2005) orbit. For the former, arc-wise initial conditions and additional empirical orbit parameters were estimated by solving the satellite equations of motion, allowing for a continuous orbit solution. The kinematic orbit, on the other hand, was determined as set of epoch-wise 3-dimensional positions in a purely geometrical way and independent of satellite dynamics (and thus constituting suitable information for subsequent Earth gravity field determination). The results shown in Bock et al. (2014) demonstrate that the PSO determination could be successfully carried out throughout the entire mission duration, with larger orbit degradations only for days with data problems, instrument calibrations, or satellite anomalies when GOCE was not flying in drag-free mode. Overall, SLR showed an accuracy of 1.84 cm for the reduced-dynamic and 2.42 cm for the kinematic PSOs, in terms of RMS (root mean square) of SLR residuals.

With increasing solar activity in 2010 and 2011 obvious problems in the GPS tracking performance became visible, showing up as systematically larger differences between the reduced-dynamic and kinematic PSOs in bands along the geomagnetic equator (Bock et al. 2014). GOCE had a sun-synchronous dusk-dawn orbit and its ascending orbit arcs crossed the equator always in the evening hours local time^1^, during which the ionosphere is known to exhibit a very pronounced equatorial ionization anomaly and other irregularities (Balan et al. 2018). Correspondingly, only the ascending arcs were significantly affected by the mentioned artifacts (Bock et al. 2014).

Due to the band-limited sensitivity of the GOCE gradiometer (Pail et al. 2011), the low-degree part of the Earth gravity field cannot be determined from gradiometry alone, but requires the addition of GPS data. Already at early mission stages it became clear that the above mentioned ionosphere-induced artifacts in the GOCE kinematic PSO positions map into thereof derived global GPS-based Earth gravity field models and lead to pronounced signatures, e.g., of geoid height differences in bands along the geomagnetic equator (Jäggi et al. 2015). With increasing ionospheric activity these problems became more obvious, both in GPS-only and in combined GPS- and gradiometer-derived GOCE gravity field models, especially in case of the time-wise approach solutions (Jäggi et al. 2015).

GOCE POD was performed using the ionosphere-free linear combination

1 $$\begin{aligned} L_\text {if}=\frac{f_1^2L_1-f_2^2L_2}{f_1^2-f_2^2} \end{aligned}$$

of the carrier phase observations $L_1$ and $L_2$ on both GPS frequencies ($f_1={1575.42}\,\hbox {MHz}$, $f_2={1227.60}\,\hbox {MHz}$), which cancels the first-order ionospheric refraction from the observation equation. Higher-order ionospheric (HOI) terms (Petit and Luzum 2010) were neglected. It was thus initially suspected that the HOI terms are non-negligible for GOCE with its special orbit configuration and that they cause the pronounced artifacts in the GPS-based orbits and GOCE gravity fields. Jäggi et al. (2015) found, however, that the inclusion of HOI terms had only a minor impact in that respect. As an alternative mitigation strategy, the authors proposed to employ a GPS data screening based on the geometry-free linear combination

2 $$\begin{aligned} L_\text {gf}=L_1-L_2, \end{aligned}$$

which, up to carrier phase ambiguities, corresponds to the total ionospheric refraction. Since the presence of the artifacts in the kinematic orbit positions could be related to high dynamics in the ionosphere, the first time derivative of $L_\text {gf}$ (approximated by epoch differences of the 1 Hz GPS data) was computed for each epoch and tracked GPS satellite, and GPS observations with $|\textrm{d}L_\text {gf}/\textrm{d}t|>{5}\,\hbox {cm/s}$ were excluded for the POD (Jäggi et al. 2015). While this screening strategy could significantly reduce the ionosphere-induced artifacts in the gravity field solutions, it caused a degradation of the orbit quality, e.g., in terms of SLR residuals or orbit overlaps. For this reason, it was decided to not apply the mentioned screening for the generation of the operational GOCE PSOs. Consequently, while up to release 4 of the time-wise approach GOCE gravity field models (EGM_TIM_RL04, Brockmann et al. 2013) the official PSOs were used, for release 5 (EGM_TIM_RL05, Brockmann et al. 2014) the kinematic orbits were computed by the Technical University of Graz, using GPS data downweighting strategies to mitigate the artifacts (Zehentner and Mayer-Gürr 2015; Zehentner 2017). These measures helped to reduce systematic errors in the GPS-derived gravity field, but the bands along the geomagnetic equator still appear in the geoid height differences, e.g., w.r.t. EGM2008 (Brockmann et al. 2014; Brockmann 2015).

After the end of the GOCE mission on 11 November 2013, the understanding of remaining artifacts, especially in gradiometer data, significantly improved (Siemes et al. 2019), and due to that ESA initiated a reprocessing campaign of the entire GOCE data in 2018. Within the GOCE HPF team the reprocessing of the PSOs was decided to be based on (i) the latest version of the Bernese GNSS Software (Dach et al. 2015), (ii) a homogeneously reprocessed time series of GPS orbits and clock corrections based on one consistent reference frame, and (iii) improved strategies to mitigate the ionosphere-induced artifacts in the kinematic PSOs and GPS-derived gravity fields, without significantly degrading the orbit quality at the same time. Regarding the GPS products, it was decided within the GOCE HPF team to use the orbits and clock corrections computed in the frame of a reprocessing campaign for the European Gravity Service for Improved Emergency Management (EGSIEM, Jäggi et al. 2019; Sušnik et al. 2020). These products are available in daily batches of 24 h length. In analogy to the generation of the operational GOCE PSOs, however, an arc length of 30 h was required in order to avoid the orbit degradation at the day boundaries and to enable the computation of orbit overlaps of consecutive days as quality measure (Bock et al. 2007). Consequently, the concatenation of the EGSIEM GNSS products to 30 h batches needed to be addressed.

The definition of a GPS data handling strategy to mitigate the impact of ionosphere-related tracking problems was part of the reprocessing task. Different GPS data screening and downweighting strategies were tested, and it was empirically found that a downweighting strategy based on the 2nd time derivative of $L_\text {gf}$, as well as the Rate of Total Electron Content Index (ROTI) yields the most convincing results (see Sect. 2.4).

In light of the currently increasing solar activity, ionosphere-related tracking problems are likely to amplify in the near future for currently active LEOs again. The herein presented methods for downweighting affected GNSS data might thus become relevant in the near future also for the POD processing of such LEOs.

This paper is organized as follows. Section 2 focuses on the reprocessing of the GOCE PSOs. After having introduced the general POD methods in Sect. 2.1, the generation of 30 h GNSS product batches from the 24 h EGSIEM batches is described in Sect. 2.2. New phase center variation (PCV) maps had to be produced for the two GOCE GPS antennas, which is discussed in Sect. 2.3. Section 2.4 focuses on the mitigation of the ionosphere-induced artifacts in the kinematic GOCE PSOs by means of improved GPS data handling strategies and presents the finally employed weighting strategy. In Sect. 2.5, the quality of the reprocessed GOCE PSOs is analyzed by different metrics, e.g., orbit overlaps and SLR residuals. Section 3 presents GPS-derived gravity field solutions based on the reprocessed GOCE kinematic PSOs. In Sect. 3.1, the used method and parametrization for gravity field recovery is described. Results are analyzed in terms of bi-monthly (Sect. 3.2) and static gravity field solutions (Sect. 3.3), focusing on the impact of the POD downweighting strategy and the additional use of GOCE accelerometer data. Section 3.4 studies the capability to recover time-variable gravity field signals from the reprocessed GOCE PSOs. Finally, Sect. 4 concludes with a summary and an outlook.

## Reprocessing of GOCE precise science orbits

In this section, we summarize the employed methods for the GPS-based GOCE POD and give some details regarding the generation of the required 30 h batches of GNSS products. Furthermore, the newly generated PCV maps, as well as the utilized GPS data downweighting strategy are presented. Finally, validation results for the reprocessed PSOs are presented and compared to the quality of the operationally generated PSOs.

**Table 1:** Processing standards used for reprocessed GOCE PSOs

Item	Reduced-dynamic	Kinematic
GPS measurement model	Undifferenced ionosphere-free phase	Undifferenced ionosphere-free phase
	igs08.atx$^{\textrm{a}}$	igs08.atx
	GOCE phase center offsets (PCOs) and variations (PCVs)	GOCE PCOs and PCVs
	EGSIEM reprocessing GPS orbits and 5 s clock$^{\textrm{b}}$	EGSIEM reprocessing GPS orbits and 5 s clock
	30 h arc length	30 h arc length
	Elevation cut-off ${0}^{\circ }$	Elevation cut-off ${0}^{\circ }$
	10 s sampling	1 s sampling
Gravitational forces	GOCO05S Earth gravity field ($200\times 200$, only static part)$^{\textrm{c}}$	
	Solid Earth tides (IERS2010)$^{\textrm{d}}$	
	Pole tides (IERS2010)$^{\textrm{d}}$	
	Ocean tides (EOT11a, $50\times 50$)$^{\textrm{e}}$	
Non-gravitational forces	No explicit modeling	
	Empirical constant accelerations per 30 h arc in radial (R)	
	tangential (T) and normal (N) direction and piecewise-constant	
	RTN accelerations at 6 min intervals and ${20}\,\hbox {nm}/\hbox {s}^{2}$ constraints	
Reference frame	ITRF2008/IGb08$^{\textrm{f}}$	ITRF2008/IGb08
	EGSIEM reprocessing Earth Rotation Parameters (ERPs)	EGSIEM reprocessing ERPs
	GOCE star tracker quaternions for attitude	GOCE star tracker quaternions for attitude
Estimation	Batch least squares	Batch least squares

$^{\textrm{a}}$Schmid et al. (2016)

$^{\textrm{b}}$Sušnik et al. (2020)

$$^{\textrm{c}}$$Mayer-Guerr (2015)

$^{\textrm{d}}$Petit and Luzum (2010)

$^{\textrm{e}}$Savcenko and Bosch (2012)

$$^{\textrm{f}}$$Rebischung (2012)

### Methods for GPS-based GOCE POD

The reprocessed (as well as the original) GOCE PSOs are computed from undifferenced GPS carrier phase observations in a Precise Point Positioning (PPP, Zumberge et al. 1997) approach, where GPS orbit positions, clock corrections and Earth rotation parameters are introduced from an external solution. This external solution was decided within GOCE HPF to be the time series of the EGSIEM GNSS reprocessing campaign (Sušnik et al. 2020), which is based on the reference frame realization IGb08 (Rebischung et al. 2012; Rebischung 2012).

The original GOCE PSO consists of a reduced-dynamic (Jäggi et al. 2006) and a kinematic (Švehla and Rothacher 2005) orbit. They are generated in one processing chain with an arc length of 30 h. The GOCE PSO generation is described in detail in Bock et al. (2007, 2011b, 2014). The strategy for the PSO reprocessing closely follows the original processing strategy, with the exception of using different GNSS input products (Sect. 2.2), partially different dynamical models, recomputed PCV maps (Sect. 2.3), and a dedicated GPS data weighting strategy to address the ionosphere-induced artifacts in kinematic PSO positions (Sect. 2.4). Table 1 gives a summary of the dynamical and measurement models used for the new reduced-dynamic and kinematic PSOs.

### Generation of 30 h GNSS products

Generally, orbit solutions are degraded close to the arc boundaries because there the orbit parameters are supported by less observations. A strategy to overcome these degradations is to process the LEO orbits in batches exceeding 24 h arc length and to then extract the 24 h batches spanning midnight to midnight. Additionally, the computation of LEO orbits in longer arcs allows for the comparison of orbit solutions of subsequent days (so-called orbit overlaps) as an internal quality measure. Therefore, both the operational, as well as the reprocessed GOCE PSOs were decided to be processed in 30 h batches, spanning from 21:00 of the previous day to 03:00 of the next day.

The generation of GOCE PSOs of 30 h arc length required the corresponding GNSS (GPS) products for the same arc length. The input GNSS products of the EGSIEM reprocessing campaign were generated in a fixed 24 h processing scheme. Hence, the GNSS orbit positions and clock corrections, as well as the Earth Rotation Parameters (ERPs) of three consecutive days needed to be concatenated. While this is straightforward for orbit positions and ERPs, the concatenation of GNSS satellite clock corrections across day boundaries needs more attention. They need, in particular, to be shifted to be continuous and connected in a phase-consistent way, and to account for the orbit misclosures at midnight (Bock et al. 2007). These clock modifications require that the daily 24 h clock corrections contain the midnight epoch for the subsequent day. This is, however, not given for the clock product of the EGSIEM reprocessing campaign. As commonly adopted for the products delivered to the International GNSS Service (IGS, Johnston et al. 2017), the last epoch is the nominal epoch prior to midnight, i.e., for the 5 s clock corrections it is 23:59:55 GPST. Due to the rather noisy nature of the GPS clocks, a straightforward extrapolation from 23:59:55 to 00:00:00 is not possible without a marked deterioration of the resulting GOCE PSOs at the day boundaries.

Therefore, in a first step, the clock corrections for the midnight epochs had to be computed from a proper clock densification from 30 s to 5 s sampling (Bock et al. 2009), using GPS data of ground stations including the midnight epoch of the subsequent day. For each day the same set of ground stations as for the creation of the EGSIEM clock corrections themselves was aimed for (in average more than 100 stations). If data were available for a given ground station on day *n*, but not on day $n+1$, this station had to be excluded for the clock densification.

**Fig. 1:**
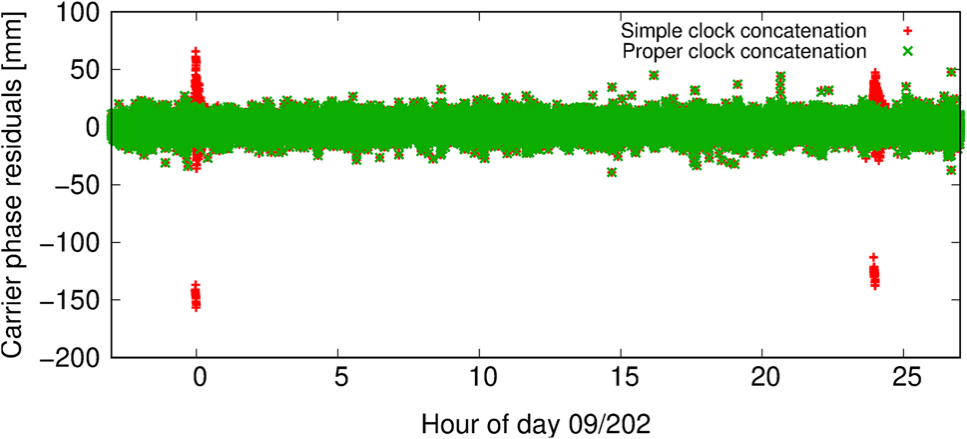
Ionosphere-free carrier phase residuals of a reduced-dynamic GOCE POD for day 09/202 (21 July 2009) when using 30 h GPS clock corrections from a simple concatenation (red) and the described proper concatenation (green). Simple clock concatenation leads to obvious degradations at the day boundaries

With the clock products extended by the midnight epoch of the subsequent day at hand the procedures for proper clock concatenation developed in the frame of the HPF activities (Bock et al. 2007) could be employed to generate high-quality 30 h satellite clock corrections. Figure 1 shows for an example day the advantage of the described clock generation over a simple concatenation in terms of carrier phase residuals of a reduced-dynamic GOCE POD.

### Phase center variation maps

Phase center variation (PCV) maps are required both for the GPS transmitting antennas, as well as for the GOCE receiving antennas. For the transmitting antennas the IGS antenna model igs08.atx (Schmid et al. 2016) was used, consistent with the reference frame realization IGb08. For the receiving antennas ground-calibrated PCV maps exist (Dilßner et al. 2006), but such maps are usually not very accurate and an in-flight determination at actual spacecraft environments is preferred due to near-field multipath effects (Montenbruck et al. 2008, 2009; Jäggi et al. 2009). For the operational generation of the GOCE PSOs PCV maps of both GPS antennas were generated by iterative stacking of carrier phase residuals of a reduced-dynamic POD over an extended time span (Bock et al. 2011a).

Prior to 2013, the IGS antenna model igs08.atx was solely based on measurements from terrestrial antennas, which limited the GPS antenna PCVs to a maximum nadir angle of ${14}^{\circ }$. Since spaceborne GPS antennas also receive signals at larger nadir angles, for the generation of the operational GOCE PSOs the GPS PCV values between ${14}^{\circ }$ and ${17}^{\circ }$ were set to constant values (equal to the values at ${14}^{\circ }$). In June 2013 (GPS week 1745) an extension of the igs08.atx model to ${17}^{\circ }$ was published, based on the inclusion of GPS data of various LEOs (Jäggi et al. 2012; Schmid 2014; Schmid et al. 2016). The reprocessing of the GOCE PSOs could thus benefit from more realistic transmitter PCVs, but, correspondingly, the GOCE PCV maps had to be recomputed.

**Fig. 2:**
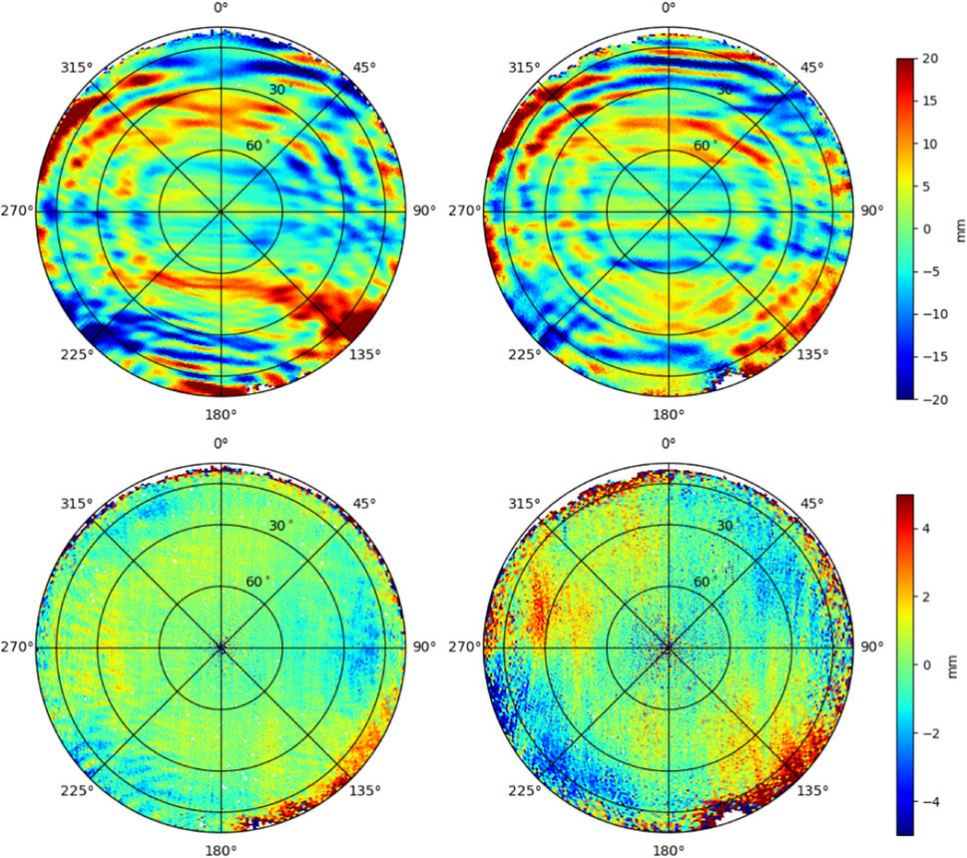
Top: New PCV map of main (left) and redundant (right) GOCE GPS antenna. Bottom: Differences of new w.r.t. old PCV maps for main (left) and redundant (right) antenna. Notice the different color scales on top and bottom

GOCE was usually tracking GPS with its main antenna. However, for certain time spans the redundant GPS antenna was in use and for days 003 and 006–041 of 2011 only the redundant antenna was active. For the PSO reprocessing it was thus necessary to re-create the PCV maps of both GOCE GPS antennas. The PCV maps were again generated by an iterative stacking of carrier phase residuals from a reduced-dynamic POD, where 10 iterations were performed (after which the carrier phase RMS values have fully converged). In each iteration, the ionosphere-free residuals were binned and averaged in $1^\circ \times 1^\circ$ bins in azimuth and elevation and these values were introduced as corrections into the next iteration. For the main antenna, residuals of 249 days between April 12 and December 31, 2009, were used. For the redundant antenna residuals of 172 days between March 26, 2010, and October 20, 2013. Figure 2 shows the new PCV maps for both antennas, as well as their differences w.r.t. the PCV maps used for the generation of the operational GOCE PSOs. For the nominal antenna, the systematic corrections are generally close to the values found in Bock et al. (2011a), with extreme values of ${-54.9}$ and 89.1 mm at very low elevations. Above ${10}^{\circ }$ elevation the extreme values read ${-27.5}$ and 26.4 mm. Larger differences w.r.t. the old PCV map are visible at low elevations and between azimuth angles of about ${90}^{\circ }$ and ${180}^{\circ }$. Above ${10}^{\circ }$ the minimum and maximum differences amount to ${-22.9}$ and 17.0 mm, and when excluding also elevations above ${80}^{\circ }$ the differences w.r.t. the old PCV map are between ${-3.3}$ and 2.6 mm. The redundant antenna correction pattern shows slightly different features than the main antenna (with extreme values of ${-98.9}$ and 105.4 mm when taking all elevations into account or ${-47.0}$ and 32.0 mm above ${10}^{\circ }$ elevation). The differences of the new w.r.t. the old PCV map are slightly larger than in case of the main antenna (between ${-29.9}$ and 46.3 mm above ${10}^{\circ }$ elevation and between ${-11.7}$ and 15.8 mm for elevations between 10 and ${80}^{\circ }$).

### GPS data weighting strategy

For the GOCE PSO reprocessing, the mitigation of ionosphere-induced artifacts in kinematic orbits and thereof derived gravity field solutions, especially the pronounced bands along the geomagnetic equator, was revisited. The goal was to find a GPS data handling strategy, which, on the one hand, reduces the systematic degradation of kinematic PSOs along the geomagnetic equator during periods of high ionospheric activity, and, on the other hand, does not degrade the general orbit quality, e.g., in terms of SLR residuals for both the kinematic and reduced-dynamic orbits.

Substantial insight into the ionosphere-related artifacts could be gained from ESA’s three-satellite magnetic field mission Swarm (Friis-Christensen et al. 2008), launched on November 22, 2013. At least at the beginning of the mission, where solar and thus ionospheric activity were relatively high, GPS-derived kinematic Swarm orbits suffered from problems very similar to GOCE (Jäggi et al. 2016). By dedicated changes of the GPS receiver settings on the different Swarm satellites, it could be shown that the problem is related to corrupted GPS data, which is produced by the GPS receivers with non-optimal settings at high ionospheric activity (van den IJssel et al. 2016; Dahle et al. 2017).

The geometry-free linear combination $L_\text {gf}$ of carrier phase observations on two GPS frequencies, Eq. (2), corresponds, up to carrier phase ambiguities and constant biases, to the total refraction which is experienced by the microwave signal when passing through the ionosphere. For GOCE, as well as for Swarm, it became soon clear that systematically deteriorated kinematic positions are linked to periods, where $L_\text {gf}$ changes rapidly and the GPS receivers are not able to properly follow the signal dynamics. Discussions with the Swarm GPS receiver manufacturers, as well as inspection of $L_\text {gf}$ during periods where kinematic orbit positions are known to be deteriorated, revealed that not the first, but actually higher time derivatives of $L_\text {gf}$ are critical and might serve as basis for advanced mitigation strategies (Schreiter et al. 2019).

Additionally, instead of omitting GPS observations which are identified as problematic, it was found to be beneficial to downweight the observations in the batch least squares adjustment of the kinematic and reduced-dynamic POD. A main reason for this is that omission may cause significant data gaps, which might require the introduction of new carrier phase ambiguity parameters, weakening the global solution.

Numerous tests were performed to assess the impact of downweighting strategies based on different time derivatives of $L_\text {gf}$ and different thresholds. Each test consisted of a reduced-dynamic and kinematic GOCE POD over a fixed time span and a subsequent gravity field recovery from the so-derived kinematic orbits (see Sect. 3). The orbit quality was assessed both in terms of internal measures (i.e., differences between reduced-dynamic and kinematic orbits, orbit overlaps), as well as by means of independent SLR validation. The resulting gravity field solutions were compared against superior Gravity Recovery and Climate Experiment (GRACE, Tapley et al. 2004) inter-satellite ranging gravity field solutions to quantify the impact of the given GPS data downweighting strategy on gravity field recovery. We refer to Schreiter et al. (2019), where similar tests have been conducted for Swarm and where details can be found on the computation of the higher time derivatives of $L_\text {gf}$.

As a second indicator, we considered the Rate of Total Electron Content (TEC) Index (ROTI, Pi et al. 1997), which is a measure for ionospheric scintillation and defined as

3 $$\begin{aligned} \textrm{ROTI}=\sqrt{\frac{\langle \varDelta \textrm{TEC}^2\rangle -\langle \varDelta \textrm{TEC}\rangle ^2}{\varDelta t^2}}, \end{aligned}$$

where $\varDelta \textrm{TEC}$ is the change of (slant) TEC within the time interval $\varDelta t$ and where the averaging takes place over 30 s. The TEC can be computed from the geometry-free linear combination $L_\text {gf}$ as

4 $$\begin{aligned} \textrm{TEC} = \frac{L_\text {gf}f_1^2f_2^2}{40.3\text {m}^{-3}\text {s}^{-2}(f_1^2-f_2^2)} \times 10^{-16}\frac{\text {TECU}}{e/\text {m}^2}+b+\epsilon , \nonumber \\ \end{aligned}$$

where the constant *b*, containing carrier phase ambiguities and biases, is irrelevant for the computation of ROTI, and where $\epsilon$ denotes the measurement noise.

Zehentner and Mayer-Gürr (2015) reported that a mitigation of the artifacts in the GPS-derived GOCE gravity field solutions could be achieved by downweighting GPS data based on the ROTI. In the same manner as for the time derivatives, tests were performed to assess different scalings of ROTI to derive GPS data weights. It was observed that the ROTI-based downweighting of GPS data is markedly less efficient for the mitigation of the artifacts along the geomagnetic equator when compared to downweighting strategies based on time derivatives of $L_\text {gf}$. On the other hand, ROTI-based downweighting allowed to significantly reduce the global noise of the GPS-derived gravity field solutions. It was thus decided to make use of ROTI as well for the downweighting strategy.

The finally (by empirical means) selected GPS data downweighting strategy can be summarized as follows:

1. For each dual-frequency GPS carrier phase observation compute the geometry-free linear combination $L_\text {gf}$ and ROTI.
2. Compute $\textrm{d}^2 L_\text {gf}/\textrm{d}t^2$, as well as the geographic latitude $\varphi$ of the GOCE satellite position.
3. If $|\textrm{d}^2L_\text {gf}/\textrm{d}t^2| >{0.04}\,\hbox {cm}/\hbox {s}^{2}$ and $|\varphi |<{50}{}^{\circ }$, set $\sigma _d = 5$, otherwise $\sigma _d=1$.
4. Set $$\sigma _r=6\cdot \textrm{ROTI}$$.
5. Set $$\sigma =\max (\sigma _d,\sigma _r)$$.

The so-determined $\sigma$ is then used to compute the weight $w=\sigma _0^2/\sigma ^2$ for the corresponding ionosphere-free phase observation $L_\text {if}$, with $\sigma _0$ the a priori uncertainty of unit weight. Figures 4 and 5 give an impression on the amount and geographic location of the data downweighted according to this strategy. Notice that apart from these weights, the data were weighted uniformly.

**Fig. 3:**
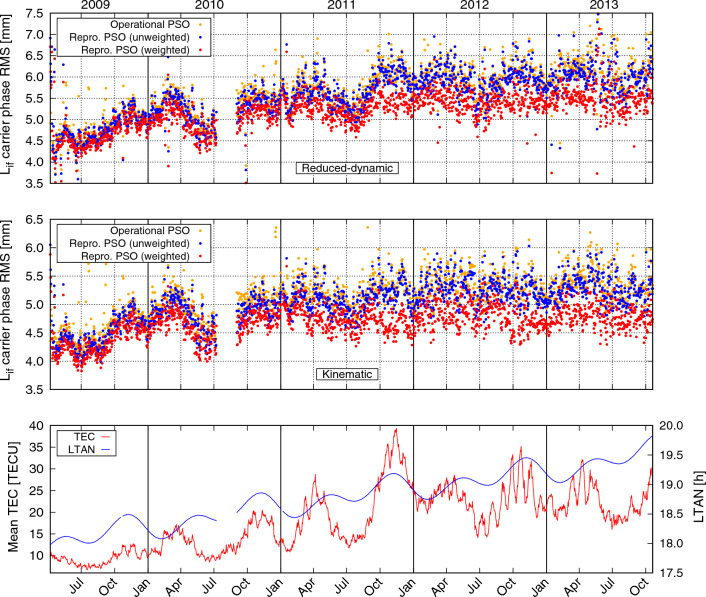
Daily ionosphere-free carrier phase RMS values for operational and reprocessed reduced-dynamic (top) and kinematic (center) GOCE PSOs. The bottom plot shows the daily mean TEC values, as well as GOCE’s local time of ascending node (LTAN). $1~\text {TECU}=10^{16}~e/\text {m}^2$

### Orbit results and validation

The GOCE orbit reprocessing comprised the time span 09/097-13/293 (07 April 2009–20 October 2013). For 1593 out of these in total 1658 days, a reduced-dynamic and kinematic orbit solution could be generated. In case of the orbits that were operationally generated, the total number of days with an orbit solution amounted to 1590. For 65 days no orbits could be generated in the reprocessing because no Level 1b data was available (62 days) or because the POD procedures failed (3 days). These latter days are 19 and 20 October 2009 (only sparse tracking data due to a satellite anomaly), and 09 June 2012 (drag-free mode reactivated after having entered safe mode on 07 June).^2^

To investigate the impact of the data downweighting strategy presented in Sect. 2.4 mainly on gravity field recovery, for the entire time span the PSOs were reprocessed once with and once without data downweighting. The reprocessed orbits finally delivered to ESA are the ones based on data downweighting. In this section, we will present results mainly for the delivered reprocessed PSOs and compare them to the metrics of the operationally generated PSOs. For the sake of convenience we call the orbits based on original GPS data simply “unweighted” and the ones computed using the data downweighting strategy presented in Sect. 2.4 “weighted”.

Figure 3 shows daily RMS values of GPS carrier phase residuals for the reduced-dynamic and kinematic PSOs for the entire time span, as well as the daily mean TEC values and GOCE’s local time of ascending node (LTAN). Besides seasonal variations of the phase residuals, a general increase of phase noise can be observed during the course of the mission, which is related to an overall increasing ionospheric activity (as indicated by the TEC values), but also due to the drift of LTAN toward later hours with larger equatorial ionospheric scintillations shortly after sunset (Balan et al. 2018). Due to the measures undertaken to counteract ionosphere-related degradations, the PSOs based on weighted GPS data show the least increase of noise. The impact of the downweighting becomes most clearly visible starting from fall 2011 on, where the mean TEC had its peak value and was remaining in average at relatively high level until the end of the GOCE mission. Table 2 collects the average RMS values for all years individually and over the entire mission. Considering the entire time span, a reduction of average carrier phase RMS by 8% and 9% for the reduced-dynamic and kinematic orbits can be reported compared to the operationally generated GOCE PSOs.

**Fig. 4:**
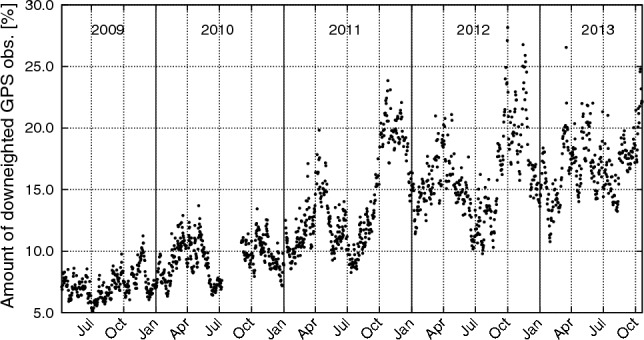
Amount of downweighted GPS data compared to total number of phase observations used for the kinematic POD at 1 Hz data rate

Figure 4 shows the daily amount of downweighted GPS observations compared to the total number of observations used for the kinematic POD (which made use of full data rate of 1 Hz). A clear correlation of this amount of downweighted data with ionospheric activity can be observed, but, interestingly, the maximum amount of data is downweighted toward end of 2012, while there the mean TEC was slightly lower than toward end of 2011 (see Fig. 3). For November 2009, 2010, 2011 and 2012, Fig. 5 displays the geographic locations of GOCE at which GPS observations used for POD were downweighted according to the strategy presented in Sect. 2.4. It can clearly be seen that observations are downweighted predominantly in bands north and south to the geomagnetic equator, and to a slightly larger extent in the region close to the South Atlantic Anomaly. Fewer observations are also downweighted over the poles. The number of downweighted observations becomes larger in the later years, cf. also Fig. 4, and it is visible that the downweighting according to the 2nd time derivative of $L_\text {gf}$ was applied only for geographic latitudes smaller than $50^\circ$ in absolute value. The number of weights due to $L_\text {gf}$ is dominant: for the four shown months they constitute 99.6%, 99.2%, 97.9% and 98.2% of the total number of weights, respectively. A close inspection also reveals that a larger number of GPS observations were downweighted very close to the geographic equator. This is attributed to the fact that the ionosphere-free linear combination $L_\text {if}$ shows large jumps (related to phase jumps on both $L_1$ and $L_2$) to an increasing degree when GOCE crossed the equator in ascending arcs. This was also observed by Guo et al. (2017), and the reason to this feature remains unexplained to us.

**Table 2:** Average carrier phase RMS values in mm of operational/(weighted) reprocessed PSOs for all years and the entire mission

Period	Reduced-dynamic	Kinematic
2009	4.85/4.64	4.60/4.34
2010	5.33/5.03	5.00/4.66
2011	5.75/5.27	5.22/4.73
2012	5.98/5.42	5.34/4.79
2013	6.12/5.46	5.38/4.79
2009–2013	5.64/5.19	5.14/4.68

For the period 2009–2013 the average carrier phase RMS values for the unweighted reprocessed reduced-dynamic and kinematic PSOs are 5.55 mm and 5.02 mm, respectively

**Fig. 5:**
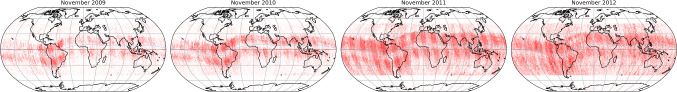
Geographic positions of GOCE at which GPS observations have been downweighted. The blue curve indicates the location of the geomagnetic equator

Due to the 30 h orbit arc length, consecutive arcs overlap for a time span of 6 h. As in Bock et al. (2014) we analyzed orbit differences within the 5 h period 21:30–02:30, i.e., excluding the last 30 min of the first arc and the first 30 min of the subsequent arc to avoid degradations at the arc boundaries. Daily overlap differences have been computed at 10 s intervals. Table 3 shows the mean values of the daily overlap RMS differences for the individual years and the entire mission duration, for both the operational and the reprocessed reduced-dynamic PSOs. Six problematic days with very large overlap differences (degraded POD due to GOCE satellite anomalies and data problems) have been excluded from the statistics. A consistent reduction of overlap RMS values can be reported for the reprocessed PSOs w.r.t. the operational PSOs for all years and directions. Especially in cross-track direction a significant reduction of the overlap RMS can be observed. Over the entire mission duration, the relative reduction in radial, along-track, cross-track and 3D amounts to 10%, 13%, 39% and 31%, respectively. The largest reduction (47%) can be reported for 2012 in cross-track direction. The reduction in overlap RMS is mostly due to smaller variations (standard deviations) and only to a small extent due to a reduction of the mean values. As an example, the average cross-track daily mean values over the entire mission duration drops from 0.4 mm for the operational PSOs to 0.1 mm for the reprocessed ones.

**Table 3:** Mean RMS in mm of daily 5 h overlap differences for the operational/(weighted) reprocessed reduced-dynamic PSOs

Period	Radial	Along-track	Cross-track	3D
2009	3.0/2.9	3.6/3.4	8.2/5.1	9.8/7.0
2010	3.0/2.7	3.5/3.0	7.3/4.5	9.0/6.3
2011	3.0/2.5	3.6/2.9	7.6/4.8	9.2/6.4
2012	2.8/2.3	3.4/2.7	8.1/4.3	9.5/5.9
2013	4.0/3.6	4.7/4.2	8.7/5.8	11.1/8.4
2009–2013	3.1/2.8	3.7/3.2	8.0/4.9	9.7/6.7

For the period 2009–2013 the corresponding values for the unweighted reprocessed reduced-dynamic PSOs read 2.9, 3.4, 4.9, and 6.8 mm, respectively

The comparison of reduced-dynamic and kinematic PSOs allows for an additional internal consistency check. Because especially the kinematic orbits are sensitive to the GPS data quality, these differences can serve in particular as a measure for data quality. Orbit positions from both types of orbits have been compared at a 10 s sampling using the central 24 h of each 30 h arc to avoid degradations at the arc boundaries. Differences with absolute values exceeding 1 m (less than 0.1%) have been omitted. Table 4 shows the mean values of the daily RMS differences for different periods and for the different directions. Again, a consistent reduction of differences for the reprocessed PSOs can be reported. Over the entire mission duration the relative reduction in radial, along-track, cross-track and 3D amounts to 5%, 8%, 6% and 8%, respectively. The largest reduction (11%) can be observed for 2011 in along-track direction. The small increase of differences for 2009 in cross-track can be attributed to the fact that the set of reprocessed PSOs is more complete than the set of operational PSOs. Neglecting the days missing for the operational PSOs in the statistics for the reprocessed PSOs as well, the mean RMS values for 2009 drop to 12.6 mm, 8.6 mm, 7.2 mm, and 16.9 mm. Notice that the statistical values in Table 4 for the operational PSOs differ slightly from the values in Tab. 2 of Bock et al. (2014), because there the differences were computed based on the entire 30 h arcs.

**Table 4:** Mean RMS in mm of daily 24 h differences between reduced-dynamic and kinematic operational/(weighted) reprocessed PSOs

Period	Radial	Along-track	Cross-track	3D
2009	13.1/12.8	8.9/8.7	7.3/7.4	17.6/17.2
2010	16.4/15.5	10.8/10.4	9.5/8.9	21.8/20.5
2011	23.8/22.5	17.3/15.4	17.8/16.9	32.4/29.9
2012	29.9/28.5	22.4/20.4	22.3/20.8	40.9/37.7
2013	35.2/33.1	27.4/25.0	26.4/24.3	49.6/45.2
2009–2013	24.1/22.8	17.7/16.2	17.1/16.0	33.0/30.5

For the period 2009–2013, the corresponding values for the unweighted reprocessed PSOs read 22.6, 16.1, 15.8, and 30.2 mm, respectively

**Table 5:** Mean and standard deviations in mm of SLR residuals for the operational and (weighted) reprocessed PSOs

Period	Operational		Reprocessed	
	Red.-dyn	Kinematic	Red.-dyn	Kinematic
2009	$-1.2\pm 17.7$	$-1.4\pm 19.1$	$1.0\pm 18.0$	$0.9\pm 19.5$
2010	$2.3\pm 16.5$	$2.3\pm 19.1$	$4.6\pm 16.9$	$4.2\pm 19.3$
2011	$1.4\pm 17.1$	$2.2\pm 25.1$	$3.1\pm 16.0$	$3.9\pm 23.4$
2012	$2.9\pm 17.8$	$4.7\pm 25.1$	$3.6\pm 17.2$	$4.9\pm 22.8$
2013	$-0.9\pm 22.8$	$1.7\pm 29.3$	$-0.1\pm 20.4$	$2.0\pm 25.6$
2009–2013	$1.3\pm 17.7$	$2.3\pm 23.5$	$2.7\pm 17.1$	$3.6\pm 21.8$

For the period 2009–2013, the corresponding values for the unweighted reprocessed reduced-dynamic and kinematic PSOs read $2.7\pm 17.3$ mm and $3.6\pm 22.1$ mm, respectively

Finally, both the operational and the reprocessed PSOs have been consistently validated using Satellite Laser Ranging (SLR), which allows for an external and independent orbit validation, and which is enabled by the 7-prism SLR retroreflector on-board GOCE. Using SLR normal point observations of the International Laser Ranging Service (ILRS, Pearlman et al. 2019), SLR residuals have been computed as differences between observed and computed ranges between GOCE and the SLR station, without estimating any correction parameters. Azimuth- and nadir-dependent range corrections for the retroreflector have been applied in nearest-prism approximation (Montenbruck and Neubert 2011). SLR station coordinates according to SLRF2014 (v20/04/28, ILRS 2017) were introduced. They were linearly extrapolated from the reference epoch (January 1, 2010) to the epoch of interest, and then corrected for postseismic deformations (PSD, ITRF 2017), the effects of solid Earth and pole tides (IERS2010, Petit and Luzum 2010), ocean tidal loading (FES2004, Lyard et al. 2006), atmospheric tidal loading (Ray and Ponte 2003) and atmospheric pressure loading (Wijaya et al. 2013). Notice that the usage of SLRF2014 station coordinates is strictly speaking incompatible to the GOCE satellite orbits being derived from IGb08-based GPS products. However, as was also observed in Arnold et al. (2019), the SLRF2014-based station coordinates are of better quality than the coordinates in SLRF2008. This can be confirmed also for the validation of the operational and reprocessed GOCE PSOs: using SLRF2014 coordinates allows to include more stations (see below) and leads to smaller SLR residuals.

For the validation, normal point data of the following 21 ILRS stations were used: Arequipa, Badary, Beijing, Changchun, Grasse, Graz, Greenbelt, Haleakala, Hartebeesthoek, Herstmonceux, Matera, Monument Peak, Mt Stromlo, Potsdam, San Fernando, San Juan, Svetloe, Tahiti, Tanegashima, Yarragadee, and Zimmerwald. An outlier threshold of 20 cm and an elevation cutoff of 10$^\circ$ was applied. In addition, a few single passes with residual RMS exceeding 3 times the station RMS were omitted. For the time span 2009–2013 about 4% of the normal points delivered by the above 21 stations were screened away. Table 5 shows the mean values and standard deviations for the operational and reprocessed PSOs for the individual years and for the entire time span. For the years 2009 and 2010, a slight increase in SLR residual standard deviations from the operational to the reprocessed PSOs can be observed, for the later years and over the entire time span a reduction of the standard deviation can be reported. To some extent, the slightly larger values can again be attributed to a larger set of reprocessed PSOs. For example, for 2009 the SLR residuals for the weighted reprocessed reduced-dynamic PSOs drop to $0.8\pm 17.7$ mm when considering only the days also contained in the set of operational PSOs. For the corresponding kinematic PSOs, the residuals drop to $0.8\pm 19.4$ mm and thus still show a slightly larger standard deviation than the operational PSOs. The corresponding set of *unweighted* reprocessed kinematic PSOs show almost identical SLR residuals of $0.8\pm 19.3$ mm, such that the slight increase of standard deviation can only marginally be attributed to the GPS data downweighting. For 2010, the unweighted reprocessed reduced-dynamic and kinematic PSOs exhibit SLR residuals of $4.6\pm 17.2$ mm and $4.5\pm 19.4$ mm, respectively, showing that for this year they actually decrease due to the GPS data downweighting.

Despite the small increase of SLR residual standard deviation in 2009 and 2010, considering the entire time span 2009–2013 a reduction of standard deviation of about 3% and 7% for the weighted reprocessed reduced-dynamic and kinematic PSOs can be reported.

## GPS-based gravity field recovery

As GPS-derived kinematic orbits provide a purely geometrical orbit solution that is independent of the LEO orbital dynamics, they are well suited to recover the long-wavelength part of the Earth’s gravity field. In this section, such a GPS-based gravity field recovery is performed in order to (i) validate the reprocessed GOCE kinematic PSOs in the gravity field domain and to (ii) provide an improved GOCE-GPS static gravity field solution that can be used as an input for combined GOCE-only gravity field models (e.g., Brockmann et al. 2021). Furthermore, we will analyze whether it is possible to recover meaningful time-variable gravity signals from the availability of about four years of GOCE GPS data.

**Table 6:** Overview of the used a priori force models and their maximum degree and order (d/o) for GPS-based gravity field recovery

Forces	Model	Max. degree
A priori gravity field	AIUB-GRACE03S$^{\textrm{a}}$	d/o 120 (static)
3rd body attractions	JPL DE421$^{\textrm{b}}$	–
Solid Earth and pole tides	IERS 2010 conv.$^{\textrm{c}}$	–
Ocean tides	EOT11a$^{\textrm{d}}$	d/o 100
De-aliasing	AOD1B RL05$^{\textrm{e}}$	d/o 100
Ocean pole tides	Model by Desai$^{\textrm{f}}$	d/o 120
Atmospheric tides	AOD1B RL05$^{\textrm{e}}$	d/o 100

$^{\textrm{a}}$Jäggi et al. (2011)

$^{\textrm{b}}$Folkner et al. (2009)

$^{\textrm{c}}$Petit and Luzum (2010)

$^{\textrm{d}}$Savcenko and Bosch (2012)

$^{\textrm{e}}$Dobslaw et al. (2013)

$$^{\textrm{f}}$$Desai (2002)

### Method for gravity field recovery

Several methods have been proposed to derive gravity field information from kinematic LEO orbit positions, see, e.g., Baur et al. (2014) for an overview. In this paper, the Celestial Mechanics Approach (CMA, Beutler et al. 2010) is applied, following the procedure described in Jäggi et al. (2015) for the processing of the GOCE operational PSOs. In the CMA, the 1-sec kinematic positions of the reprocessed GOCE PSOs (clipped to 24 h) are used as pseudo-observations to solve a generalized orbit determination problem as implemented in a development version of the Bernese GNSS Software.

In a first step, an observation screening of the kinematic orbit positions with respect to the reduced-dynamic orbits is performed in order to remove outliers with a 3D-difference larger than 0.15 m. In a second step, an initial orbit determination is carried out to generate a priori orbits on a daily basis. For this purpose, the kinematic positions are weighted according to their epoch-wise covariance information and are fitted over 24-hour arcs by a numerical integration of the equation of motion based on the a priori force models indicated in Table 6.

In addition to the six initial Keplerian osculating elements, the following arc-specific empirical and stochastic parameters are set up in order to absorb non-gravitational forces: (i) constant and once-per-revolution empirical accelerations in the radial (R), tangential (T), and normal (N) directions acting over the daily arc-length, and (ii) pseudo-stochastic pulses (instantaneous velocity changes) in R/T/N directions with a spacing of 6 min that are weakly constrained (0.1 mm/s) to avoid singularities in case of data gaps. Note that in contrast to the GOCE POD in Sect. 2.1, pulses are used as stochastic parameters instead of accelerations, following the argumentation in Jäggi et al. (2015).

Besides a purely empirical treatment of non-gravitational accelerations by means of the aforementioned empirical and stochastic orbit parameters, the measured GOCE common-mode accelerometer data (GOCE EGG_CCD product, ESA 2008) can be introduced to the gravity recovery process. The benefit of this will be studied in Sect. 3.3.

The gravity field recovery from the kinematic positions is accomplished in a third step, in terms of a generalized orbit improvement. The actual orbits are linearized around the computed a priori orbits and are expressed as truncated Taylor series with respect to the unknown arc-specific orbit and gravity field parameters, i.e., spherical harmonic (SH) coefficients up to a specific degree and order (d/o). Based on the partial derivatives of the Taylor series, daily normal equations (NEQs) are set up according to standard least-squares adjustment. After the pre-elimination of arc-specific parameters, daily NEQs are accumulated into NEQs spanning longer time periods and inverted to solve for the corrections of the SH coefficients with respect to the used a priori gravity field model.

Based on the described concept, the kinematic positions of the reprocessed GOCE PSOs have been used to generate bi-monthly and static gravity field solutions that will be analyzed and discussed in the following sections. For the assessment of these solutions, we use the superior gravity field model ITSG-Grace2018 (Kvas et al. 2019) as a reference, which is based on independent and ultra-precise GRACE inter-satellite ranging data. Comparisons are made (i) in the spatial domain by global geographical grids of geoid height differences and (ii) in the spectral domain by difference degree amplitudes. As the estimation of the zonal and near-zonal SH coefficients are strongly affected by the polar gap of the GOCE orbit configuration (inclination close to ${96.5}^{\circ }$), these coefficients are excluded for the analyses of difference degree amplitudes according to the rule of thumb by van Gelderen and Koop (1997), i.e., SH coefficients with $m< \left( {6.5}^{\circ } \cdot n \cdot \pi /{180}^{\circ }\right)$ are not included, where *n* and *m* are the SH degree and order, respectively. For the evaluation of geoid height differences, a Gaussian filter with a radius of 300 km is applied to the SH coefficients (cf. Wahr et al. 1998), in order to focus on the long- to medium-wavelength part of the differences. To prevent that comparisons are affected by secular trends of the Earth’s time-variable gravity field, we use the linear trends provided by ITSG-Grace2018 to propagate its static part to the reference epoch of the GOCE data, i.e., the middle of the respectively processed time interval. However, in the case of shorter time spans, we make use of the monthly ITSG-Grace2018 solutions (Mayer-Gürr et al. 2018).

**Table 7:** Number of daily orbit arcs used for generating GOCE GPS-based bi-monthly gravity field solutions

	2009	2010	2011	2012	2013
Jan–Feb	–	37	38	56	46
Mar–Apr	–	56	58	55	61
May–Jun	–	58	59	48	46
Jul–Aug	–	(5)$^\textrm{a}$	60	61	58
Sep–Oct	–	28	58	59	45
Nov–Dec	58	59	58	59	–
Total	58	243	331	338	256

$^\textrm{a}$Only used for static gravity field solutions in Sect. 3.3

### Bi-monthly gravity field solutions

In a first step, we will analyze how the use of the reprocessed GOCE PSOs and the downweighting strategy impacts the gravity field recovery. According to the 61-days repeat cycle of the GOCE initial science orbit, we use the kinematic positions of the reprocessed PSOs to compute (adjacent) bi-monthly GPS-based gravity field solutions up to d/o 120 without applying any regularization and without the use of accelerometer data. In total, 23 bi-monthly solutions have been generated covering the entire science mission phase between Nov 2009 and Oct 2013, where only the Jul–Aug period of 2010 is missing due to a major satellite anomaly in 2010 (cf. Floberghagen et al. 2011). For each bi-monthly solution, Table 7 indicates the number of included daily orbit arcs. Most of the missing days can be attributed to events of various anomalies as specified in the GOCE End-of-Mission Operations report (ESA 2014). Additionally, some of the daily arcs were excluded after manual inspection of the residuals of the orbit fits generated in step two of the gravity field determination.

**Fig. 6:**
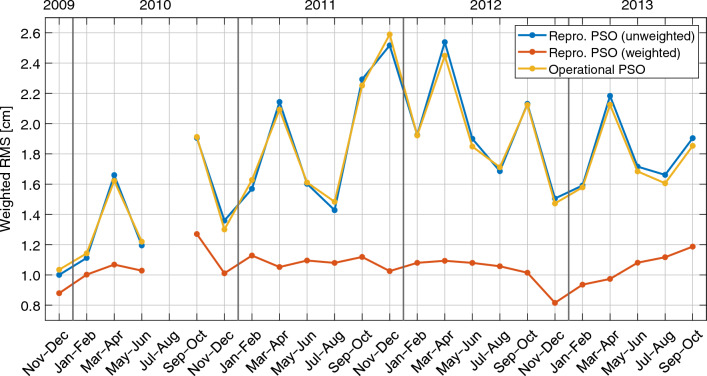
Weighted RMS values of geoid height differences with respect to ITSG-Grace2018 of bi-monthly GOCE GPS-based gravity field solutions covering the entire science mission phase between Nov 2009 and Oct 2013. RMS values are plotted for solutions based on the unweighted and weighted kinematic positions of the reprocessed PSOs, blue and red curve, respectively, as well as the kinematic positions of the operational PSOs (yellow curve). Note that a 300 km Gaussian filter is applied to the geoid height differences and that all RMS values refer to the region with latitudes $|\varphi |<{50}^{\circ }$

To get an impression of the quality of the bi-monthly gravity field solutions and how it changed during the mission lifetime, Fig. 6 provides geographically weighted RMS values of geoid height differences with respect to monthly ITSG-Grace2018 fields. In line with the GPS downweighting strategy presented in Sect. 2.4, the RMS values are restricted to the region with latitudes $|\varphi |<{50}^{\circ }$. In Fig. 6, results are presented for solutions based on the unweighted and weighted kinematic orbits of the reprocessed GOCE PSOs by the blue and red curve, respectively. For comparison, the yellow curve shows RMS values for a gravity field recovery based on the operational GOCE PSOs (taken from Jäggi et al. 2015). Note that the comparability is limited to a small extent as a priori force models and included daily arcs are not exactly consistent.

By comparing the blue and yellow curve in Fig. 6, it is clearly visible that for all bi-monthly solutions the gravity field recovery based on the operational PSOs and the unweighted reprocessed PSOs provide an almost identical quality. The differences in their respective RMS values are below the 1 mm level, where no general systematics can be observed. This demonstrates that both types of kinematic orbits provide a comparable performance from the perspective of gravity field determination. This validates the (unweighted) reprocessed PSOs and also illustrates that the updated GPS products and new PCV maps that were used for the orbit generation do not significantly impact the gravity field recovery, which could be expected.

**Fig. 7:**
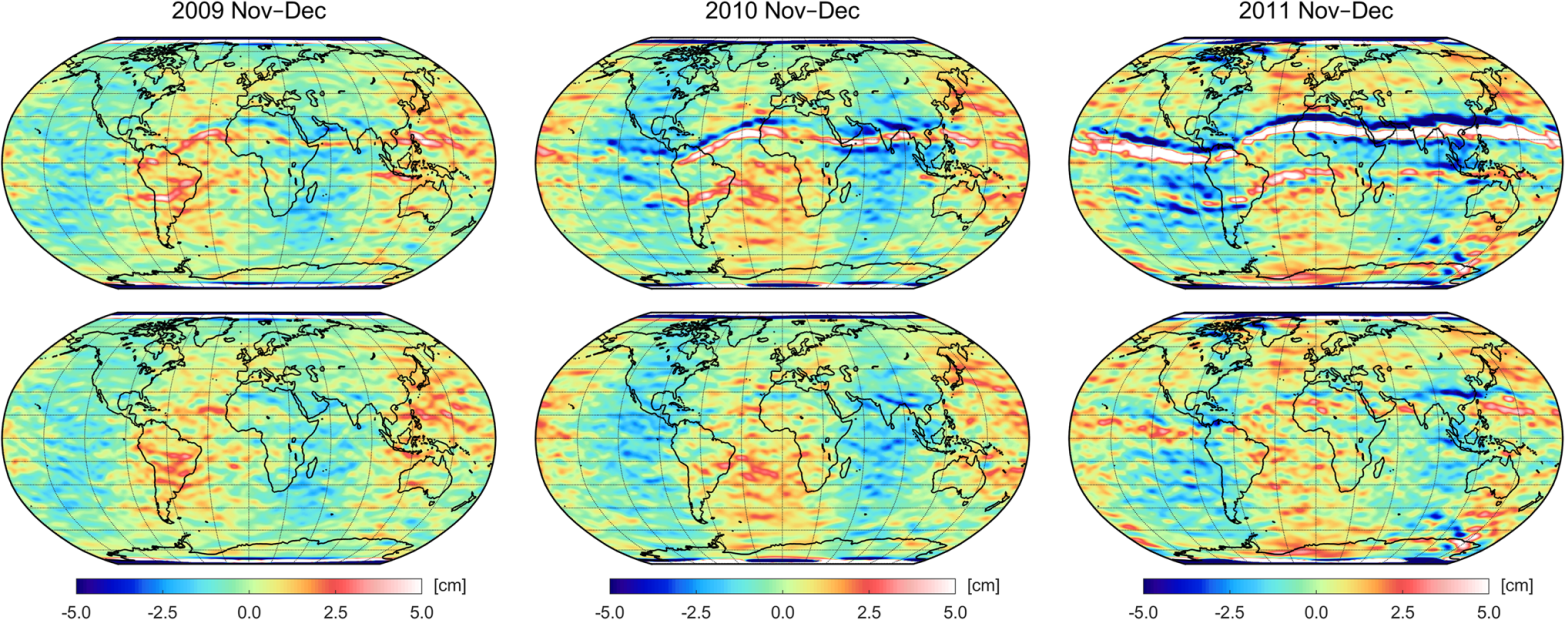
Geoid height differences with respect to ITSG-Grace2018 of bi-monthly GOCE GPS-based gravity field solutions based on the unweighted and weighted kinematic positions of the reprocessed PSOs, top and bottom row, respectively, for the November–December period of the years 2009 (left), 2010 (middle), and 2011 (right column). Note that a 300 km Gaussian filter is applied

The RMS values obtained by the unweighted reprocessed orbits show a strongly varying quality, where the lowest value of 1.00 cm is reached for Nov–Dec 2009 and the largest value of 2.53 cm is reached for Mar–Apr 2012. Comparing the variations of the blue curve with the ones of the mean TEC values in Fig. 3 (bottom) provides an indication that the quality of the bi-monthly solutions is strongly affected by the changing ionospheric activity during the GOCE mission lifetime.

A completely different picture can be seen when considering the red curve representing the use of the weighted reprocessed orbits, where the downweighting strategy is applied in order to account for ionospheric-induced effects. Compared to the other two solutions, the RMS values of the geoid height differences are considerably reduced and within a range of 0.5 cm around a mean value of 1.1 cm. This impressively demonstrates that gravity field recoveries based on the weighted reprocessed orbits have a strongly improved and much more consistent quality level, where no correlation between the RMS values and the ionospheric activity is visible.

To confirm that the improvements seen in Fig. 6 are related to a reduction of ionospheric-induced errors, Fig. 7 shows geoid height differences of the bi-monthly solutions for the Nov–Dec period of the years 2009, 2010, and 2011. These solutions were chosen exemplarily as they represent different quality levels according to Fig. 6. In the top row of Fig. 7, results are plotted for the use of the unweighted reprocessed orbits. It can be seen that all gravity fields are affected by systematic artifacts located in bands north and south of the geomagnetic equator. In accordance with the RMS values shown in Fig. 6, the magnitudes of these artifacts increase year by year, where the strongest impact with magnitudes up to 20 cm can be detected for the solution of the Nov–Dec period of 2011.

In the bottom row of Fig. 7 geoid height differences are shown for the solutions based on the weighted reprocessed orbits. In all three cases, the gravity field recoveries show remarkable reductions of the systematic signatures along the geomagnetic equator. While the artifacts are almost completely removed in the case of 2009 and 2010, some signatures with strongly reduced amplitudes are still visible for 2011. According to Fig. 6, these improvements result in an impressive reduction of the weighted RMS of about 12%, 26%, and 59% for the Nov–Dec period of the years 2009, 2010, and 2011, respectively. For all geoid height differences shown in Fig. 7, alternating long-wave patterns are prominently visible, indicating discrepancies in the low-degree sectorial coefficients. As already reported in Jäggi et al. (2015), these effects can mainly be attributed to the coefficient $S_{22}$ and will be addressed later on.

**Fig. 8:**
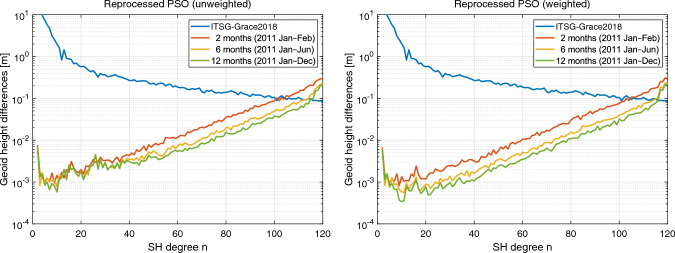
Difference degree amplitudes in terms of geoid heights with respect to ITSG-Grace2018 of accumulated gravity field solutions for two (red curve), six (yellow curve), and 12 months (green curve) of the year 2011 based on the GOCE kinematic positions of the reprocessed PSOs with and without making use of the downweighting strategy (right and left panel, respectively). Note that zonal and near-zonal coefficients are excluded according to van Gelderen and Koop (1997)

In the next step, we analyze the effect of the reprocessed GOCE PSOs on the generation of gravity fields covering longer periods. For this purpose, we take the most affected year 2011 as an example. In Fig. 8, difference degree amplitudes are shown for gravity field solutions based on the kinematic positions of the reprocessed PSOs for two (red curve), six (yellow curve), and 12 months (green curve) of the year 2011. In the left panel of Fig. 8, results are plotted for the unweighted kinematic positions. Here, it is impressively shown, how the ionospheric-induced systematic errors affect the accumulation of data from longer time periods. For the spectral range between degree 40 and 110, the six and 12 months solutions show reduced difference degree amplitudes compared to the bi-monthly solution. However, for the most relevant lower degrees, the quality of the three solutions are on a comparable level, illustrating that the degradation due to systematic errors prevents any improvements. In contrast, in the case of a gravity field recovery based on the weighted reprocessed PSOs, as depicted in the right panel of Fig. 8, the benefit of data accumulation can be observed for almost the whole spectrum. Akin to Fig. 7, the difference degree amplitudes in Fig. 8 also demonstrate remaining errors in the lowest degrees, which we will analyze in the next section.

**Fig. 9:**
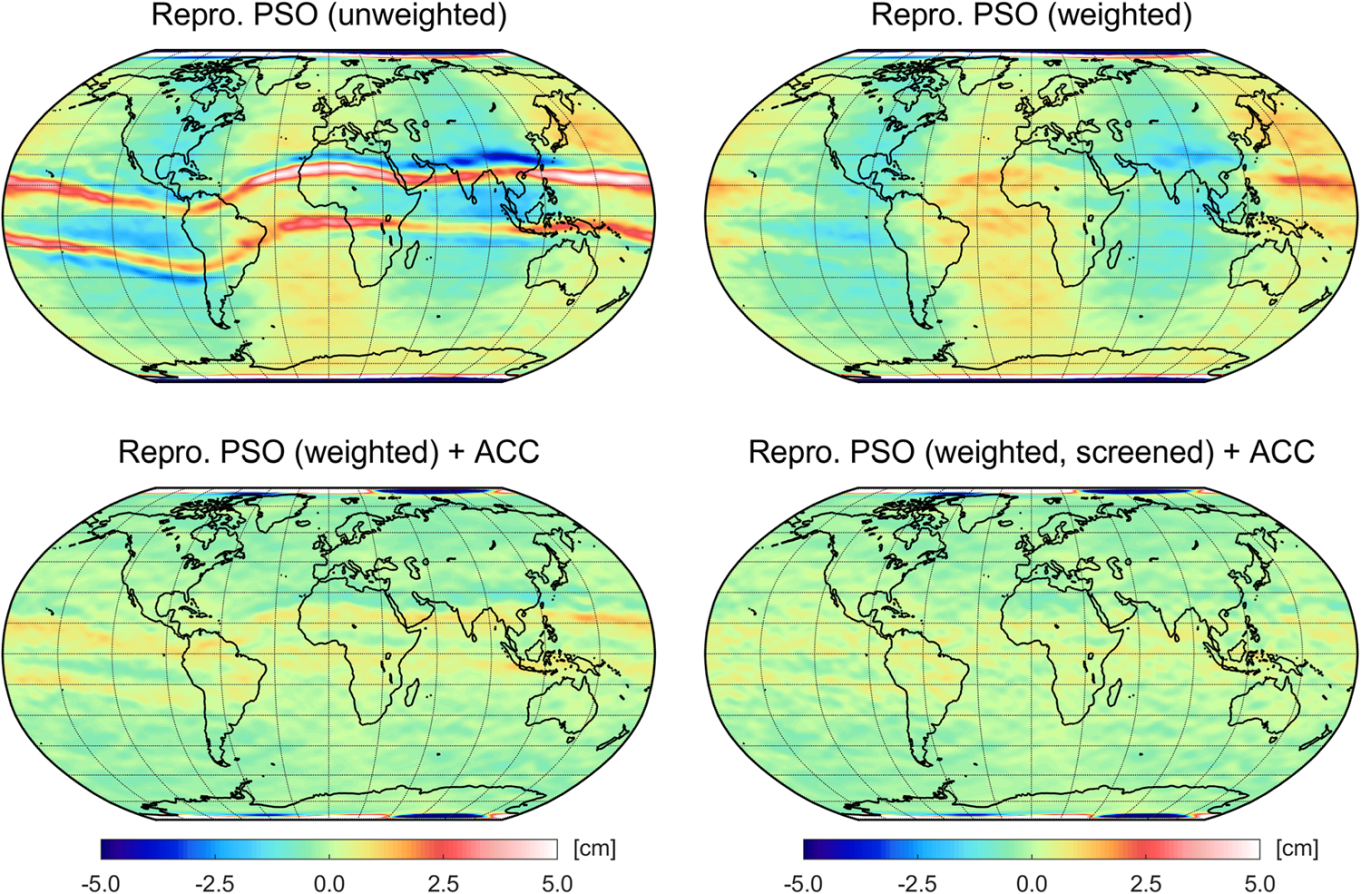
Geoid height differences with respect to ITSG-Grace2018 of GOCE GPS-derived static gravity field solutions (Nov 2009–Oct 2013) based on the unweighted and weighted kinematic positions of the reprocessed PSOs, top left and top right, respectively. Solutions shown at the bottom are based on the weighted kinematic positions taking into account accelerometer data (bottom left) and applying an additional variance-based screening (bottom right). Note that a 300 km Gaussian filter is applied

**Fig. 10:**
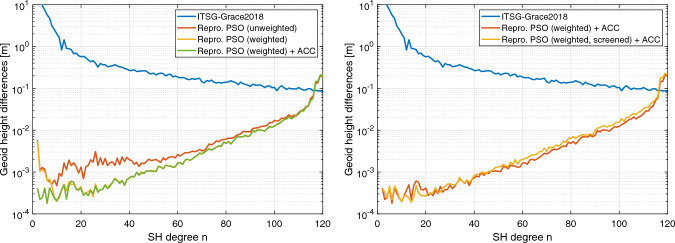
Difference degree amplitudes in terms of geoid heights with respect to ITSG-Grace2018 of GOCE GPS-based static gravity field solutions (Nov 2009–Oct 2013). On the left side, results are shown for the unweighted kinematic positions (red curve) as well as the weighted kinematic positions of the reprocessed PSOs without and with taking into account accelerometer data (ACC), yellow and green curve, respectively. On the right side, solutions are based on the weighted kinematic positions, taking into account accelerometer data (red curve) and applying an additional variance-based screening (yellow curve). Note that zonal and near-zonal coefficients are excluded according to van Gelderen and Koop (1997)

### Static gravity field

In the following, static gravity field solutions for the entire GOCE science mission phase (Nov 2009–Oct 2013) are examined that are based on the processing of 1226 (out of 1450 possible) daily arcs (cf. Table 7). Compared to the bi-monthly solutions in Fig. 7, artifacts centered along the geomagnetic equator become more pronounced and localized in the static solution based on the unweighted kinematic positions, plotted in Fig. 9 (top left). When using the weighted kinematic positions of the reprocessed PSOs (Fig. 9, top right), a remarkable reduction of these systematic signatures is achieved. The strong impact of the downweighting strategy is also reflected in substantially reduced difference degree amplitudes as shown in Fig. 10 (left side). Particularly, the SH coefficients between degree 10 and 40 strongly benefit from the weighting. No improvements are visible for degrees above 110 where the static solutions are dominated by omission error and the lowest degrees that still show larger discrepancies with respect to ITSG-Grace2018.

For improving the limited quality of low-degree SH coefficients, Jäggi et al. (2015) analyzed the additional use of the measured GOCE common-mode accelerometer data (GOCE EGG_CCD product, ESA 2008) for the GPS-based gravity field recovery. For this purpose, they introduced the accelerometer data at NEQ level in terms of binned values that were used for the modeling of additional constant accelerations acting over ${6}~\hbox {min}$ time intervals. By this procedure, Jäggi et al. (2015) demonstrate a positive impact on the gravity field recovery, which, however, was confined to the SH coefficients of degree 2. Therefore, we will examine the impact of including the accelerometer data already at the level of the observation equations as part of the a priori force model. This is certainly a more rigorous way and allows to benefit from the full accelerometer signal. Regarding parametrization, scale factors are not estimated for the accelerometer data as they are assumed to be stable and close to one (cf. Bouman et al. 2011). Remaining offsets in the accelerometer data are absorbed by the daily estimated constant empirical accelerations.

By using the weighted kinematic positions, Fig. 9 (bottom left) illustrates the impact of the accelerometer data on the static solution in the spatial domain. In comparison with the solution without accelerometer data, shown in Fig. 9 (top right), it becomes evident that the degradation causing the alternating long-wave patterns in the geoid height differences is effectively removed. The difference degree amplitudes in Fig. 10 (left side) allow to see which SH coefficients are actually affected by the use of the accelerometer data. While the solutions with and without accelerometer data (green and yellow curve, respectively) are consistent for mid to high degrees, Fig. 10 demonstrates that the GOCE accelerometer data can contribute to an improved quality of the GPS-based gravity field recovery up to degree 10. The same holds true for solutions based on the unweighted reprocessed PSOs (not shown). Compared to the procedure used in Jäggi et al. (2015), this underlines the advantage of using accelerometer data as part of the a priori force model to improve the handling of non-gravitational accelerations.

**Table 8:** Weighted RMS values of geoid height differences with respect to ITSG-Grace2018 of GOCE GPS-based static gravity field solutions covering the entire science mission phase (Nov 2009–Oct 2013) and their improvement rate (IR) in terms of percentage reduction of the RMS values with respect to the first solution

Solution	WRMS [cm]	IR [%]
Repro. PSO (unweighted)	1.18	
Repro. PSO (unweighted) + ACC	1.02	13.6
Repro. PSO (weighted)	0.67	43.2
Repro. PSO (weighted) + ACC	0.35	70.3
Repro. PSO (weighted, screened) + ACC	0.26	78.0

Note that a 300 km Gaussian filter is applied to the geoid height differences and that all values refer to the region with latitudes $|\varphi |<{50}^{\circ }$

To achieve a further reduction of the artifacts along the geomagnetic equator, we analyze an additional screening of the weighted kinematic positions of the reprocessed PSOs. This procedure is motivated by some (remaining) problematic kinematic positions, which have very large covariance values but still do not seem to be downweighted enough to significantly impact the gravity field recovery. To counteract this, a variance-based screening with an empirically determined threshold of 7 mm is applied to the kinematic positions in the region with latitudes $|\varphi |<{50}^{\circ }$, meaning that positions with larger standard deviations are excluded from the gravity field processing. Note that in contrast to the screening of GPS measurements carried out in Jäggi et al. (2015), the screening purposed here does not affect the orbit quality and is linked to the applied downweighting strategy.

As can be detected from the geoid height differences in Fig. 9, the variance-based screening enables a further significant reduction of remaining artifacts, particularly in regions of the Pacific Ocean south of Japan and Hawaii. Furthermore, due to the screening, the former streamlined signature of the artifacts is only barely visible in the gravity field. Corresponding improvements in the difference degree amplitudes (Fig. 10, right side) can be observed for degrees between 10 and 20. It should be noted that the reduced number of observations causes slightly larger difference degree amplitudes in some spectral ranges, particularly in the noise-dominated higher degrees.

To quantify the achieved improvements, Table 8 specifies weighted RMS values of the geoid height differences of the static gravity field solutions analyzed in this section. While solutions based on the unweighted reprocessed PSOs provide RMS values above 1 cm, they are well below this level in the case of the weighted reprocessed PSOs. The impact of the downweighting strategy leads to a reduction of the RMS value of about 43% (without using accelerometer data) and 66% (with accelerometer data). This discrepancy indicates a limited benefit of the accelerometer data when using the unweighted reprocessed PSOs. Furthermore, Table 8 reveals that the additional screening leads to an improved RMS value of about 26%.

### Time-variable gravity field

Finally, we focus on the capability to recover the long wavelength part of the Earth’s time-variable gravity field from the reprocessed GOCE PSOs. For this purpose, we estimate SH coefficients of the static gravity field up to d/o 120, and simultaneously solve for trends and annual variations of the time-variable gravity field up to d/o 10. As demonstrated in Jäggi et al. (2015), this can be done by a linear parameter transformation at the level of the already existing daily NEQs, such as the NEQs corresponding to the derived static gravity field solutions.

Assuming the unknown SH coefficients associated with the daily arc *i* at epoch $t_i$ are denoted by $x_{nm}^{\,i}\in \{S_{nm}^{\,i},\,C_{nm}^{\,i}\}$, the transformation then reads

5 $$\begin{aligned} \begin{aligned} x_{nm}^{\,i}&= a_{nm}^{\,i} \cdot \left[ \omega \left( t_i-t_0\right) \right] + b_{nm}^{\,i} \cdot \sin {\left[ \omega \left( t_i-t_0\right) \right] }\\&\quad + c_{nm}^{\,i} \cdot \cos {\left[ \omega \left( t_i-t_0\right) \right] } + d_{nm}^{\,i}, \end{aligned} \end{aligned}$$

where $a_{nm}^{\,i}$, $b_{nm}^{\,i}$, $c_{nm}^{\,i}$ are the amplitudes of the trend, sine and cosine term, respectively, $\omega$ denotes the annual frequency, $t_0$ is the reference epoch and $d_{nm}^{\,i}$ an offset.

**Fig. 11:**
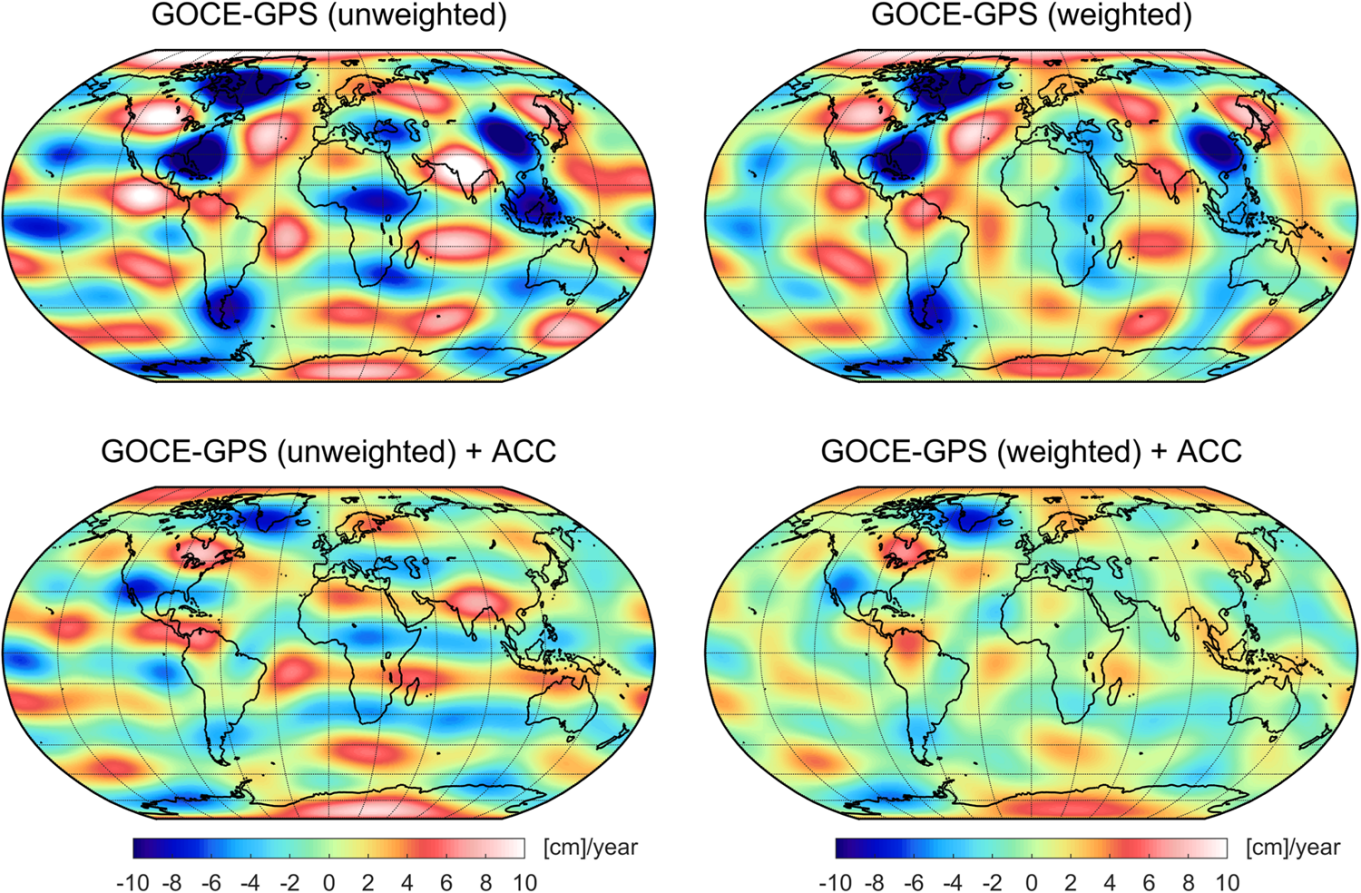
Estimated linear trends expressed in terms of equivalent water heights recovered from the GOCE reprocessed PSOs covering the entire science mission phase (Nov 2009–Oct 2013). Results are plotted for the unweighted and weighted kinematic positions, left and right, respectively, as well as, without and with using accelerometer data (ACC), top and bottom, respectively. Note that degree-2 coefficients are not included and a 300 km Gaussian filter is applied

The recovered time-variable gravity signals are visualized in terms of equivalent water heights (EWH, Wahr et al. 1998), applying a 300 and 1500 km Gaussian filter for the trends and the annual variations, respectively, and excluding degree-2 coefficients. For different approaches, Fig. 11 displays the geographical distribution of the estimated trend terms. In the top of Fig. 11, results are plotted based on the unweighted and weighted kinematic PSOs (left and right, respectively), without using accelerometer data. In both cases, the recovered trends are strongly dominated by disturbances with large amplitudes that prevent to observe reasonable time-variable signals. As visible from Fig. 11 (bottom), the results change considerably, when taking into account accelerometer data for the gravity field recovery. This is consistent with the findings in Sect. 3.3, where a positive effect of including accelerometer data has been shown for the lower degrees up to 10, i.e., the same spectral range used here for the time-variable gravity field recovery. Disturbances in the estimated trends in Fig. 11 (bottom) are significantly mitigated, making it possible to detect major mass trends in the continental areas. Furthermore, differences due to the used orbit type (weighted or unweighted PSOs) become more evident. In the case of the unweighted PSOs (Fig. 11, bottom left), remaining disturbances are more pronounced, also revealing signatures along the geomagnetic equator. In contrast, the estimated trends based on the weighted PSOs (Fig. 11, bottom right) do not show such systematics and provide an overall reduced noise level over the oceans (where no time-variable signal is expected). Note that the variance-based screening introduced in the last section leads to a slightly worse performance (not shown). Thus, the estimated temporal variations based on the weighted PSOs and accelerometer data are used for the following analysis.

**Fig. 12:**
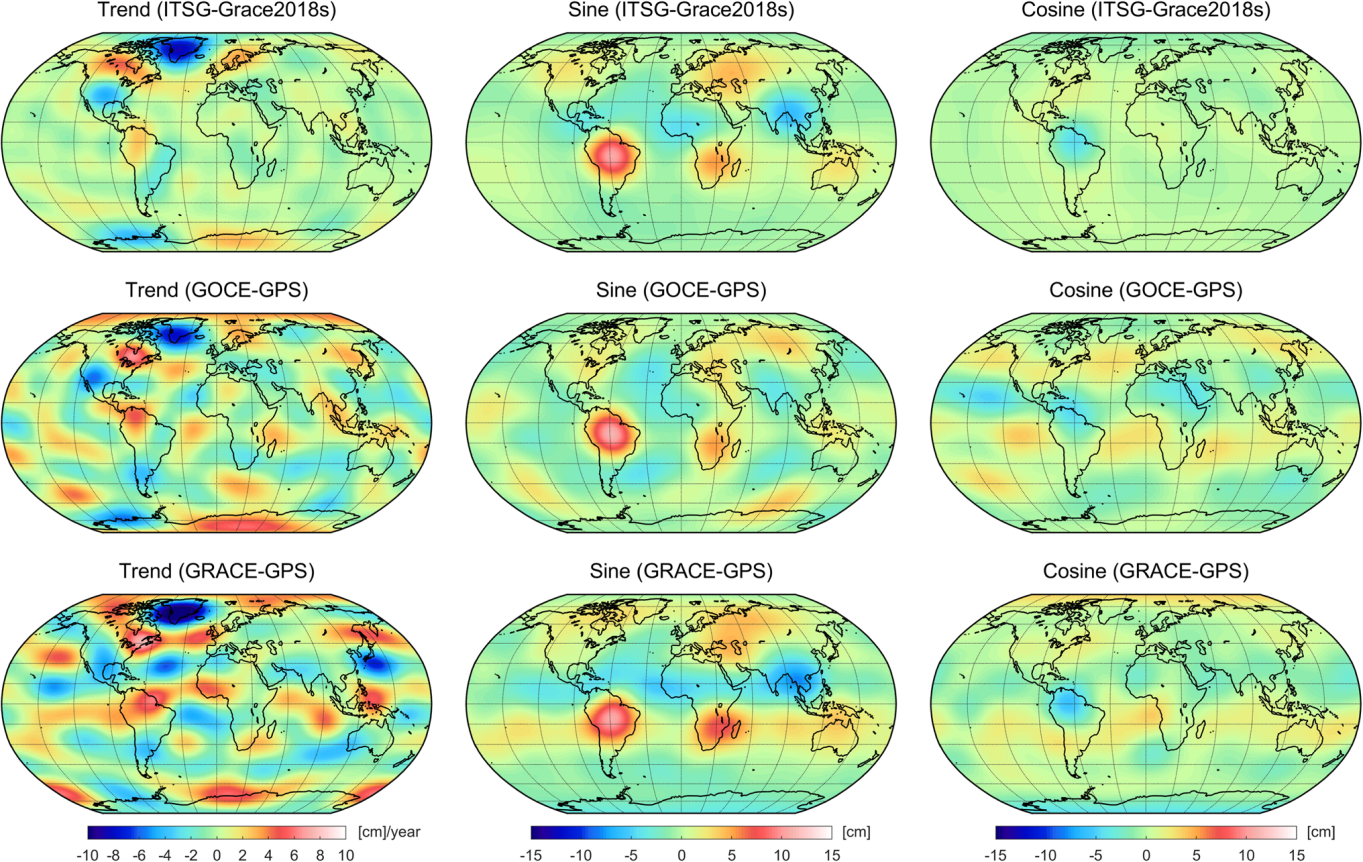
Linear trends (left) and annual amplitudes of the sine (middle) and cosine (right) component expressed in terms of equivalent water heights for the GOCE mission period (Nov 2009–Oct 2013). The results are recovered from an a posteriori fit of monthly ITSG-Grace2018 gravity field solutions (top row) or co-estimated from GOCE and GRACE GPS data, middle and bottom row, respectively. Note that the degree-2 coefficients are not included and a 300 and 1500 km Gaussian filter is applied for the trends and the periodic annual signals, respectively

To assess the quality of estimated trends and annual variations, superior reference values are derived from an a posteriori fit of the monthly ITSG-Grace2018 solutions covering the GOCE mission period (Fig. 12, top row). Besides time-variable gravity signals derived from the GOCE GPS data in this study (Fig. 12, middle row), we also include results derived from GRACE GPS data (Grombein et al. 2022) for comparison (Fig. 12, bottom row). Moreover, to quantify the performance of the GOCE and GRACE GPS-based estimates, Table 9 provides weighted RMS differences with respect to ITSG-Grace2018 reference values.

The trends recovered from ITSG-Grace2018 are dominated by strong mass changes in the polar regions (e.g., ice mass loss in Greenland and West-Antarctica or ice mass gain in East-Antarctica) and show effects of the post-glacial rebound (e.g., in Canada and Scandinavia). These major features can also be detected in the GPS-based trends although the signal amplitudes tend to have stronger magnitudes. Larger discrepancies to ITSG-Grace2018 can be observed over the oceans, where the GOCE solution is less affected by noise. This is also reflected in Table 9, where GOCE provides a 32% smaller RMS value compared to GRACE.

The sine and cosine terms illustrate the annual variations with maximum values in spring–fall and winter–summer, respectively. The predominate signals are visible for the sine term, where the seasonal peak related to hydrological variations in the Amazon river basin is most striking. Here, all three solutions provide a quite consistent shape and magnitude. Most other features are either slightly overestimated (e.g., the positive signal in South Africa in the case of GRACE) or underestimated (e.g., the negative signal in South Asia in the case of GOCE).

The signal of the cosine term as recovered from ITSG-Grace2018 is mostly confined to the Orinoco river basin in the north of South America. Although both GPS-based solutions also show this peak, they exhibit significantly more signal over the continental areas and particularly over the oceans. However, in contrast to the findings in Visser et al. (2014) and Jäggi et al. (2015) that were based on the operational GOCE PSOs, it is possible to recover a meaningful cosine term form the reprocessed PSOs in this study. For both annual variations, the RMS differences in Table 9 indicate a higher consistency between the GRACE-GPS solution and ITSG-Grace2018, i.e., RMS values are 17% and 21% smaller compared to GOCE-GPS.

**Table 9:** Weighted RMS values of equivalent water height differences of GOCE and GRACE GPS-based time-variable gravity field recoveries with respect to reference values obtained from an a posteriori fit of monthly gravity field solutions based on GRACE inter-satellite ranging data

	Trend [cm/year]	Sine [cm]	Cosine [cm]
GOCE-GPS	1.26	1.30	1.43
GRACE-GPS	1.86	1.08	1.13

Note that degree-2 coefficients are not included and a 300 and 1500 km Gaussian filter is applied for the trends and the periodic annual signals, respectively

In total, the GOCE and GRACE GPS-based time-variable gravity signals reveal a comparable quality. In the case of the recovered trends, results based on GOCE GPS data provide a better performance with a reduced noise level over the oceans. On the one hand, this might be expected due to the higher sensitivity of the lower flying GOCE satellite. On the other hand, the GOCE GPS data is much more affected by disturbances, e.g., due to the ionospheric activity. Moreover, it could be demonstrated that taking into account the accelerometer data is crucial to exploit the full potential of the time-variable gravity field signal captured by the GOCE satellite.

## Summary and conclusions

In the frame of the GOCE reprocessing campaign initiated by ESA in 2018, the AIUB was responsible for the reprocessing of the PSO product. In the first part of this paper we have presented the employed POD methods, with a particular focus on the generation of the 30 h GNSS products, the re-generated GPS antenna PCV maps and the GPS data weighting strategy to address ionosphere-related problems in kinematic orbits. We then have shown validation results for the reprocessed PSOs and have compared them to consistently generated results for the operationally processed PSOs. Overall, an improved performance for the reprocessed PSOs could be reported in terms of carrier phase RMS values, overlap differences, consistency between reduced-dynamic and kinematic PSOs, and SLR residuals. It has been shown that, overall, for the phase RMS values, the orbit overlaps and the SLR residuals the GPS data downweighting is slightly beneficial, and for the consistency between reduced-dynamic and kinematic PSOs only slightly detrimental (but nevertheless resulting in better metrics compared to the operational PSOs).

In the second part of the paper, we have analyzed the reprocessed kinematic PSOs in the light of GPS-based gravity field recovery. While the GOCE core instrument for gravity field measurements was a three-axis gravity gradiometer, the band-limited sensitivity of this instrument requires the addition of GPS data for resolving the low-degree part of the Earth gravity field. In the past, GPS-based GOCE gravity field solutions were often markedly affected by ionosphere-induced artifacts along the geomagnetic equator, related to GPS receiver tracking problems. This also affected earlier combined GPS- and gradiometer-derived GOCE gravity field models, especially in case of the time-wise approach solutions.

From the perspective of GPS-based gravity field recovery, it can be confirmed that the POD downweighting strategy presented in this paper is capable to considerably reduce ionosphere-induced artifacts along the geomagnetic equator. Compared to the quality of previous (bi-monthly) GOCE GPS-based gravity fields that was strongly varying according to the ionospheric activity, solutions based on the reprocessed PSOs provide a substantially improved and much more consistent quality level. In particular, the spherical harmonic coefficients between degree 10 and 40 strongly benefit from the weighting. Remaining issues in the low-degree coefficients of GOCE GPS-only solutions (in particular $S_{22}$), could be solved by making use of the measured GOCE common-mode accelerometer data. Here, it has been found that the accelerometer data can contribute to improved coefficients up to degree 10.

By analyzing global RMS values of geoid height differences with respect to a superior inter-satellite ranging solution, the achieved improvements can be summarized as follows. In comparison with the use of the unweighted reprocessed GOCE PSOs, the impact of the weighting strategy results in reduced weighted RMS values of geoid height differences of about 43% (without using accelerometer data) and 70% (with accelerometer data), when considering a static gravity field solutions covering the entire GOCE mission period. For a further reduction of ionosphere-induced signatures, we have proposed an additional variance-based screening of the reprocessed GOCE PSOs. By excluding kinematic positions with standard deviations larger than 7 mm from gravity field recovery, the reduction of the RMS value is increased to even 78%.

Moreover, we have demonstrated that it is possible to derive meaningful time-variable gravity field signals from the reprocessed GOCE PSOs, in terms of trends and annual variations up to d/o 10. In particular for the trend estimation, it is crucial to take into account the GOCE accelerometer data in the gravity field recovery. Compared to results based on GRACE GPS data (from the same time period), GOCE-derived trends provide a higher consistency with inter-satellite ranging solutions and a smaller noise level over the oceans. On the other hand, annual variations derived from GRACE GPS data show a slightly better performance compared to GOCE GPS data.

The good performance of the reprocessed weighted PSOs has led to the decision to use them for the generation of the release 6 of the time-wise approach GOCE-only gravity field model EGM_TIM_RL06 (Brockmann et al. 2021). The benefit of the GPS data downweighting to reduce the artifacts along the geomagnetic equator could also be confirmed in this combined GPS- and gradiometer-derived gravity field solution.

Although the GOCE mission has already been completed in 2013, the question of a proper treatment of ionospheric disturbances affecting the LEO orbit and gravity field quality will be of increasing importance in the upcoming years. The rising activity of the current solar cycle will require advanced mitigation techniques for the processing of operational LEO satellites like, e.g., Swarm. As proposed in Schreiter et al. (2019) or in this study, a weighting of affected GPS observations is most effective and should be preferred compared to a data screening strategy, e.g., used in Jäggi et al. (2016) or Dahle et al. (2017). To this end, further studies are required to determine LEO-dependent settings for a suitable downweighting of affected GPS data.

## Data Availability

All GOCE Level 1 data used for the PSO reprocessing are publicly available at ESA Earth Online, https://earth.esa.int/eogateway/catalog/goce-level-1. The GNSS products of the EGSIEM reprocessing campaign can be downloaded from http://ftp.aiub.unibe.ch/REPRO_2015. The reprocessed GOCE PSOs are available at https://earth.esa.int/eogateway/catalog/goce-level-2.

## Footnotes

1 GOCE’s local time of ascending node changed from about 18:00 on April 7, 2009 to about 19:50 on October 20, 2013 (see Fig. 3), while the beta angle, i.e., the angle of the Sun above the orbital plane, varied between 54$^\circ$ and 88$^\circ$ in the same time span.

2 See the monthly GOCE L1b Data Quality Control Reports available at https://ftp-qras.earth.esa.int/goce/egg/monthly/.
